# Malaria elimination and the need for intensive inter-country cooperation: a critical evaluation of regional technical co-operation in Southern Africa

**DOI:** 10.1186/s12936-024-04891-5

**Published:** 2024-02-28

**Authors:** Chadwick H. Sikaala, Bongani Dlamini, Alphart Lungu, Phelele Fakudze, Mukosha Chisenga, Chishala Lukwesa Siame, Nyasha Mwendera, Dumisani Shaba, John M. Chimumbwa, Immo Kleinschmidt

**Affiliations:** 1 SADC Malaria Elimination Eight Secretariat, Windhoek, Namibia; 2 Department of Infectious Disease Epidemiology, London School of Hygiene and Tropical Medicine, London, UK; 3 School of Pathology, Faculty of Health Sciences, University of Witwatersrand, Johannesburg, South Africa

**Keywords:** Malaria, Elimination, Regional collaboration, Angola, Botswana, Eswatini, Mozambique, South Africa, Namibia, Zambia, Zimbabwe, Border malaria

## Abstract

**Background:**

Malaria elimination requires closely co-ordinated action between neighbouring countries. In Southern Africa several countries have reduced malaria to low levels, but the goal of elimination has eluded them thus far. The Southern Africa Development Community (SADC) Malaria Elimination Eight (E8) initiative was established in 2009 between Angola, Botswana, Eswatini, Mozambique, Namibia, South Africa, Zambia, and Zimbabwe to coordinate malaria interventions aiming to eliminate malaria by 2030. Cross-border coordination is important in malaria elimination settings as it strengthens surveillance, joint planning and implementation, knowledge exchange and optimal use of resources. This paper describes how this collaboration is realized in practice, its achievements and challenges, and its significance for malaria elimination prospects.

**Methods:**

The ministers of health of the E8 countries oversee an intergovernmental technical committee supported by specialist working groups consisting of technical personnel from member countries and partner institutions. These technical working groups are responsible for malaria elimination initiatives in key focus areas such as surveillance, vector control, diagnosis, case management, behaviour change and applied research. The technical working groups have initiated and guided several collaborative projects which lay essential groundwork for malaria elimination.

**Results:**

The E8 collaboration has yielded achievements in the following key areas. (1) Establishment and evaluation of malaria border health posts to improve malaria services in border areas and reduce malaria among resident and, mobile and migrant populations. (2) The development of a regional malaria microscopy slide bank providing materials for diagnostic training and proficiency testing. (3) A facility for regional external competency assessment and training of malaria microscopy trainers in collaboration with the World Health Organization. (4) Entomology fellowships that improved capacity in entomological surveillance; an indoor residual spraying (IRS) training of trainers’ scheme to enhance the quality of this core intervention in the region. (5) Capacity development for regional malaria parasite genomic surveillance. (6) A mechanism for early detection of malaria outbreak through near real time reporting and a quarterly bulletins of malaria incidence in border districts.

**Conclusions:**

The E8 technical working groups system embodies inter-country collaboration of malaria control and elimination activities. It facilitates sustained interaction between countries through a regional approach. The groundwork for elimination has been laid, but the challenge will be to maintain funding for collaboration at this level whilst reducing reliance on international donors and to build capacities necessary to prepare for malaria elimination.

## Background

The sixteen members of the Southern Africa Development Community (SADC) include countries with the highest malaria burden in the world. Whilst all SADC countries have the goal of eventually eliminating malaria, currently only Lesotho, Mauritius and Seychelles are malaria-free [1].

In 2009 SADC Ministers of Health (MOH) dedicated member countries to the goal of malaria elimination by establishing a multi-country regional initiative, consisting of the eight southernmost malaria endemic countries in the region—Angola, Botswana, Eswatini, Mozambique, Namibia, South Africa, Zambia, and Zimbabwe—called the Elimination 8 Initiative (E8). The E8 was established on the premise that no one country can eliminate malaria without coordinated efforts with its neighbours because of transboundary malaria between countries. Cross-border coordination is important in malaria elimination settings as it strengthens surveillance, joint planning and implementation, knowledge exchange and optimal use of resources [2]. The vision of the E8 was to eliminate malaria in four low-transmission ‘frontline countries’—Botswana, Eswatini, Namibia, and South Africa—by 2020, and to pave the way for elimination in four moderate to high-transmission ‘second line countries’- Angola, Mozambique, Zambia, and Zimbabwe—by 2030 [3]. The formation of the E8 initiative was in line with the 2008 World Health Organization (WHO) recommendation on elimination which stresses the need for strong collaboration and political will amongst countries forming regional elimination blocks which could support countries nearing elimination. A key focus of elimination strategies would be cross border transmission [4].

**Fig. 1:**
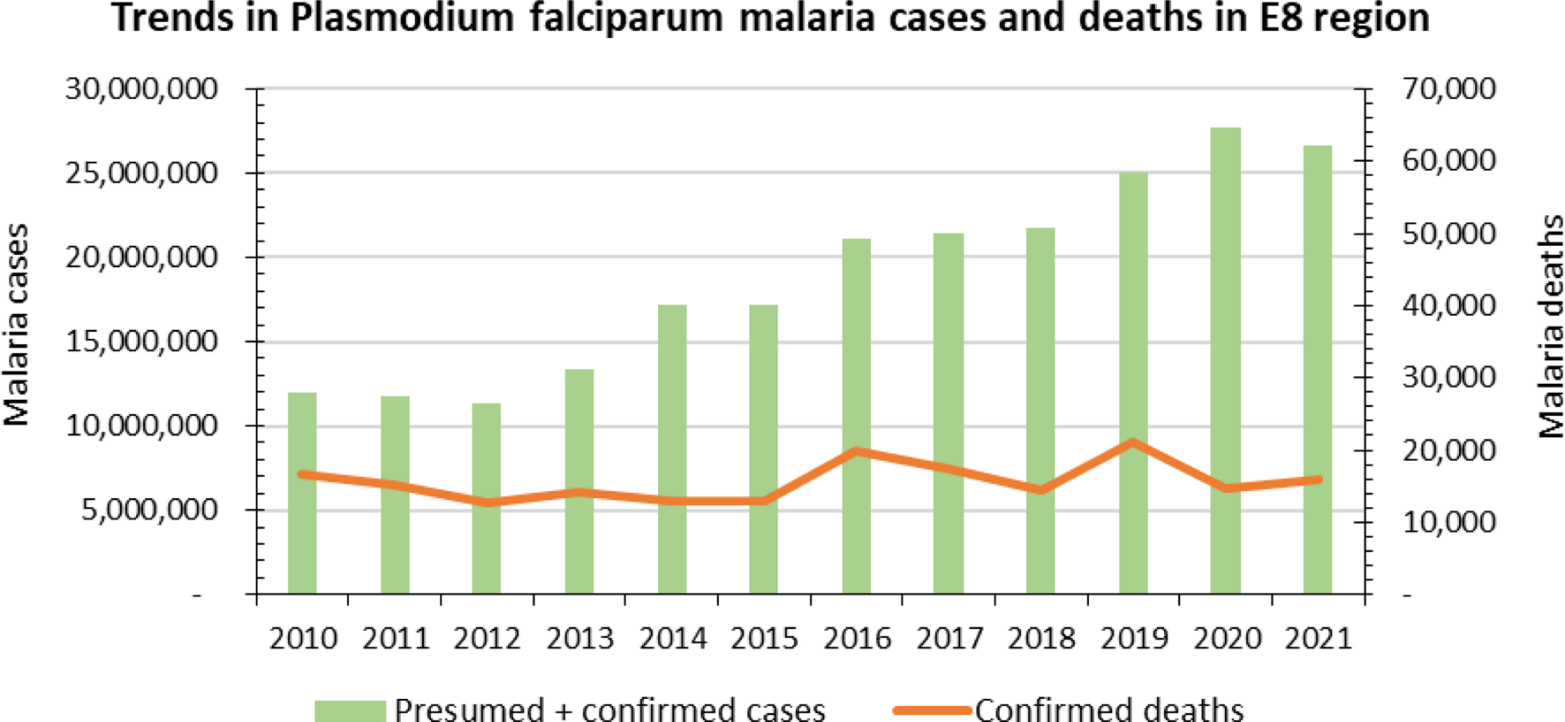
Malaria Trends across the E8 Region 2010–2021. Source. Statistics adapted from the WHO World Malaria Report 2022 statistical tables

Three of the eight countries (Angola, Mozambique, and Zambia) contributed about 98% of *Plasmodium falciparum* malaria cases and 96% of malaria deaths during the decade from 2010 to 2021, underscoring the large heterogeneity of the malaria burden in the region [5]. From 2016, the region overall, experienced a gradual increase in malaria cases (Fig. 1), thus moving further away from the goal of zero malaria, although trends varied considerably between countries (Fig. 1).

**Fig. 2:**
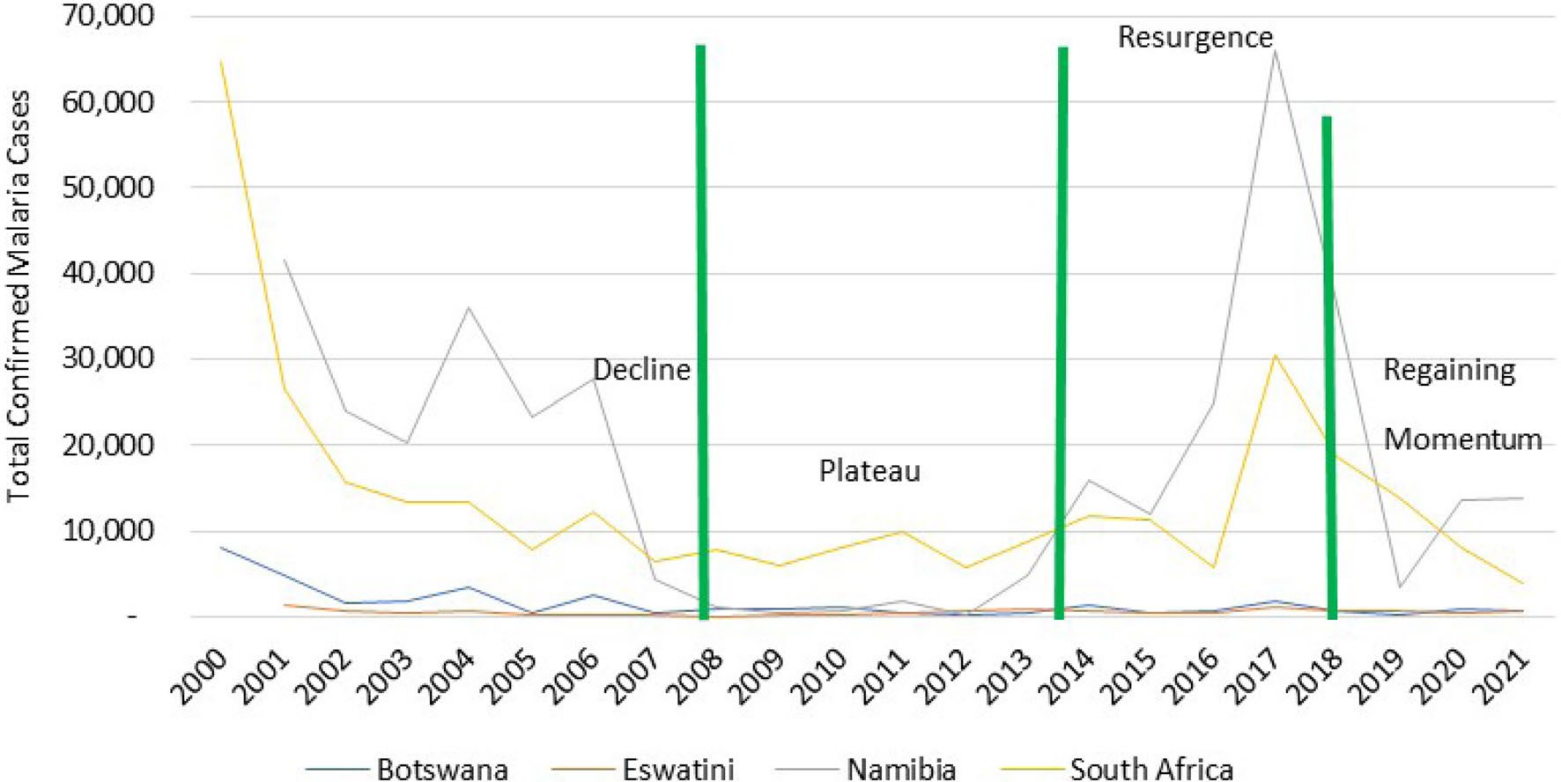
Progress towards malaria elimination in the frontline countries. Source. Statistics adapted from the World Malaria Report 2021 and E8 Scorecard 2013–2021

Frontline countries experienced sharp declines in malaria cases from 2000 to 2008, with case numbers remaining low up to 2014 (Fig. 2). Beginning in 2015, the region experienced a series of malaria outbreaks which have resulted in malaria resurgence, even where the possibilities of achieving elimination seemed to be in sight, particularly in Botswana, Eswatini and South Africa (Fig. 2). The malaria case resurgences can be attributed to, among other things, suboptimal coverages and quality of vector control, particularly indoor residual spraying and slow response where reactive case detection is practiced. In some areas, heavy rains caused population displacements and potentially increased malaria vector densities and receptivity, exposing vulnerabilities in epidemic response systems.

To regain momentum towards elimination, E8 facilitated the coordination of intensified efforts to identify and address systemic and persistent challenges, including cross-border planning to optimize deployment of interventions. This resulted in malaria cases in frontline countries declining from 99,658 in 2017 to 18,990 cases in 2021, putting malaria elimination back on track in these countries [6].

Despite this progress in frontline countries, the aim of elimination is continuously thwarted by imported cases from neighbouring second line countries. The region faces increased challenges of cross-border movement and interconnectedness which is common in many parts of sub-Sahara Africa [7]. The direction of migration is mainly from high burden second line countries to low burden first line countries primarily for economic reasons such as, seeking work and trade, and in some instances for medical treatment. A large proportion of short-term cross-border travel is circular, driven by family and community ties across [8]. This challenge is compounded by the lack of dedicated resources for malaria elimination in low transmission border districts of second line countries due in part to prioritization of funds towards higher burden districts in the north of these countries.

In response to malaria outbreaks and escalating cases, E8 Member States in 2018 re-committed themselves to strengthening inter-country cross-border collaboration by adopting the Windhoek Declaration to Elimination of Malaria [9].

This paper describes the way regional technical collaboration in malaria control and elimination has been implemented in southern Africa in practice, what it has achieved thus far and what challenges it is likely to face in future.

## Methods

### E8 governance structure and technical working groups

**Fig. 3:**
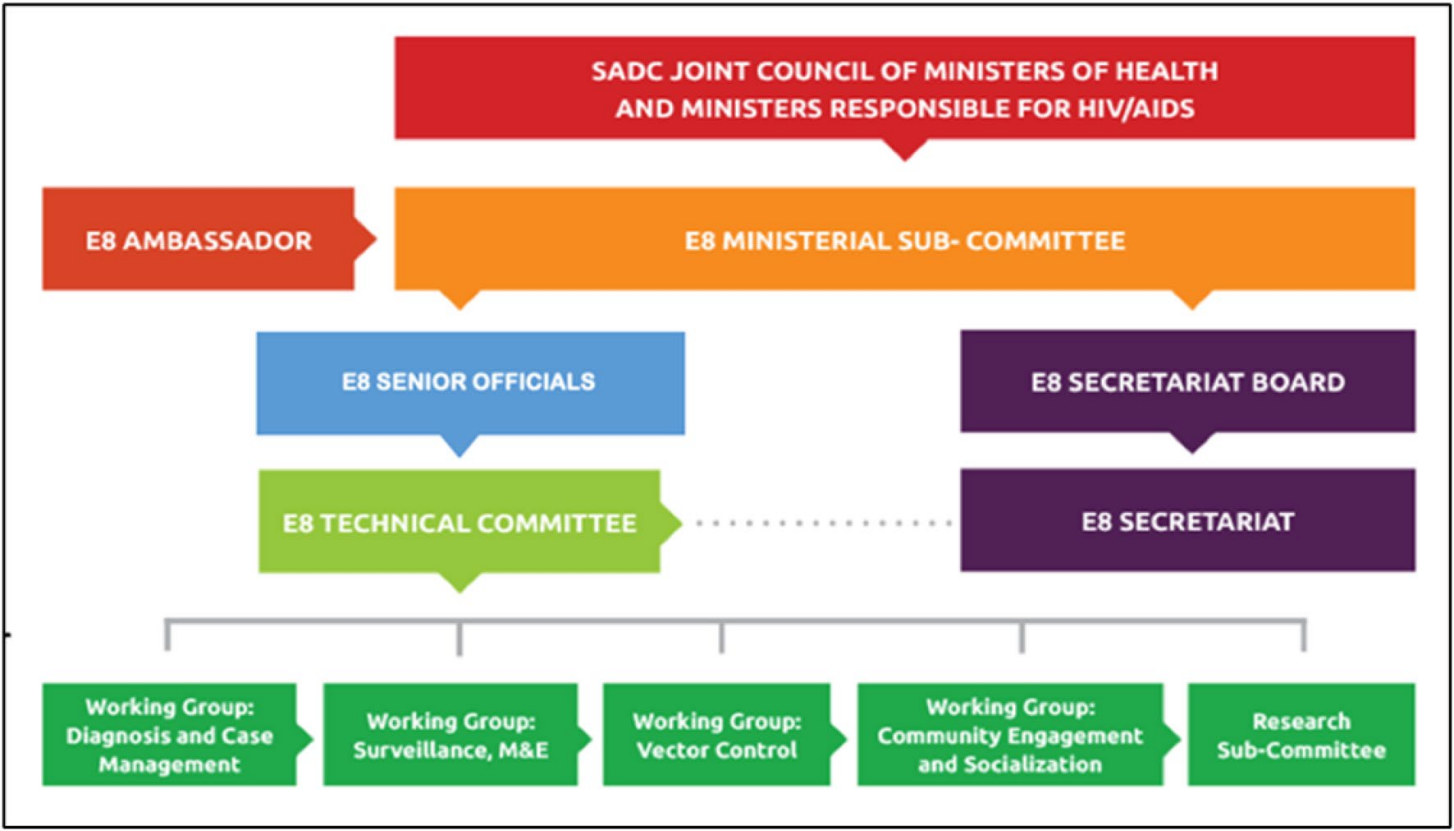
Governance structure of the Elimination Eight Initiative. Source. SADC-E8 website

The E8 is governed by the ministers of health of the E8 member countries, acting as a subcommittee of the SADC Ministerial Council of Ministers of Health, supported by the E8 Technical Committee which consists of senior officials of health of member countries, and specialized technical working groups [10]. The E8 ministerial committee provides strategic oversight and leads diplomatic dialogue at ministerial level on behalf of the Member States. The E8 is supported by a Secretariat, which co-ordinates technical collaboration amongst E8 countries (Fig. 3) and convenes the working groups which review, plan and coordinate malaria elimination strategies across the region The secretariat is responsible for translating regional resolutions into action.

The technical committee consists of the managers of all E8 national malaria control/elimination programmes; it facilitates the implementation of regional malaria control and elimination initiatives, and it oversees the specialist technical working groups.

### E8 technical working groups

These are. (i) diagnosis and case management; (ii) surveillance, monitoring, and evaluation (SME); (iii) vector control and entomological surveillance (VC); (iv) community engagement and social behaviour, change and communication; and (v) a research sub-committee.

Each technical working groups consists of representatives from each of the member states with subject expertise relevant to the thematic area. Representatives of partner organizations (e.g., WHO) also attend these meetings. The technical working groups support implementation of regional malaria elimination activities through a coordinated multi-country malaria elimination approach. They convene biannually to consider new developments in their subject areas, and advance decisions and action points, subject to endorsement by the technical committee. The secretariat coordinates approved actionable activities and coordinates meetings. Actions requiring high-level engagement and advocacy are escalated to the Senior Officials and the E8 Ministerial Committee. The technical working groups provide strategic and technical guidance and inter-country harmonization of activities within their respective thematic areas in line with WHO formative guidance and agreed regional best practices.

The SME technical working group facilitates the coordination of malaria surveillance, data sharing and emergency response to malaria outbreaks and the dissemination of standard indicators of regional significance as reported by individual member countries. The VC technical working group develops and disseminates guidance of best practices in vector control and entomological surveillance and provides a platform to identify specific regional needs in vector control and entomology. The diagnosis and case management thematic area focuses on matters related to case management guidelines, therapeutic drug resistance monitoring and capacity development in diagnostics. The community engagement and social behaviour, change and communication technical working group facilitated the regional harmonization of community engagement interventions focussing on marginalized populations along borders. The research sub-committee identifies key regional research priorities in malaria elimination, promotes operational research and scrutinizes evidence on which policies in support of elimination are based. These roles are defined in each of the technical working group terms of reference. The priority projects initiated by the E8 technical working groups since their inception in 2016 are highlighted below.

## Results

### Border health posts and malaria services in border areas

**Fig. 4:**
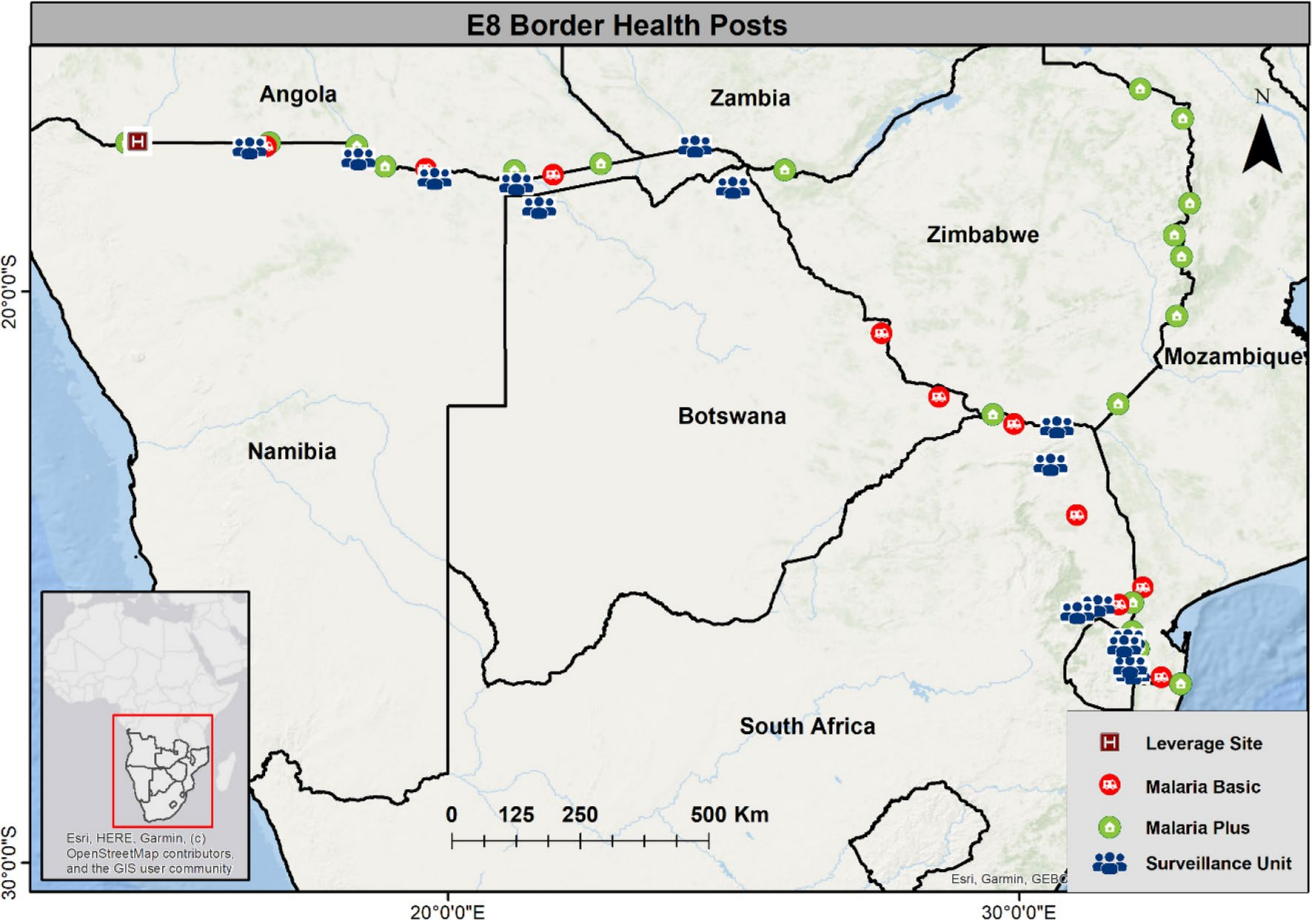
Location of Border Health Posts along the five borders in the E8 region

Due to the high interconnectedness of populations in the region, transit routes of MMPs are likely to lead to cross-border importation of parasites from high to low endemic countries thereby thwarting elimination efforts in the latter. To reduce cross-border malaria and to improve the provision of malaria services in border areas, 46 sites were identified along borders in 2017 between high and low transmission countries where border health posts were considered by national malaria programmes to have optimal impact [8, 11]. Three types of health post were deployed (Table 1), located along five key international borders shown in Fig. 4. All border posts were able to diagnose and treat malaria. Malaria plus units were accommodated in refurbished storage containers and able to additionally provide basic primary health care. Malaria basic units were mobile and provided malaria testing and treatment from a tent, whilst the Surveillance units provided malaria services from a vehicle. All units were staffed by a qualified nurse who was generally assisted by a Community Health Worker or an Environmental Health Officer. A small number of existing health facilities, called leverage border posts, were incorporated into this scheme.

**Table 1:** Border Health Posts allocation per country

Country where the border health posts are situated	Type of border health posts and numbers per country		
	Malaria plus	Malaria basic	Surveillance unit
Angola	7	1	0
Botswana	0	2	2
Eswatini	0	0	2
Mozambique	8	2	0
Namibia	1	3	4
South Africa	0	3	4
Zambia	1	1	0
Zimbabwe	4	1	0
Totals N = 46	21	13	12

The purpose of these malaria border health posts (BHP) was to improve access to malaria prevention, diagnosis, and treatment among underserved border communities and mobile migrant populations (MMP).

Between 2017 and 2020 approximately 1.26 million people were tested for malaria at these BHPs of which, about 76,000 (6%) tested positive and were given first line anti-malarial treatment in accordance with local treatment guidelines [12]. In 2020, these BHPs were transferred from the E8 secretariat to the health system of the country in which they were located. Whilst the BHPs have made malaria services more accessible to MMPs and residents in border areas, it has proved challenging for countries to fully fund these facilities from existing health budgets.

Following the establishment of the BHPs, the E8 commissioned a study to gain insight into malaria service delivery in border areas, and to assess knowledge, attitudes and practices related to malaria and the functionality and role of the health posts. The assessment was carried out through cross sectional surveys among residents and MMPs in selected sites in border areas in Angola, Botswana, Mozambique, Namibia, South Africa, Zambia, and Zimbabwe [8].

This assessment found that there was a high-level of knowledge of the causes, symptoms, and prevention of malaria amongst most respondents, that nearly all those who reported a positive blood test result when seeking care received medication, and that most but not all border residents had access to primary prevention against malaria in the form of insecticide treated nets or indoor residual spraying. Most respondents said that when they presented at health facilities with fever, they were tested for malaria. However, in areas of low endemicity respondents were not always offered a blood test for malaria when presenting with malaria symptoms (fever). Travel across borders was frequent and often associated with sleeping outside without protection against infective mosquito bites. The results were reported in detail to each of the NMCPs in participating countries, and remedial action was undertaken where appropriate [8]. This work was overseen by the research subcommittee and the SME technical working group.

### Capacity building and research

#### Malaria microscopy slide bank and external microscopy competency assessment

Since the WHO requires malaria microscopy as a confirmatory diagnostic method during the process of malaria elimination certification [13], it is essential that regional capacity for accurate microscopy is created in the E8 region. Through the diagnosis and case management working group a Malaria Slide Bank (MSB) was established as a regional facility in 2018. The MSB was set up at the National Institute for Communicable Diseases (NICD), South Africa, where it is currently accommodated, maintained, and administered. The MSB constitutes a collection of reference slides of known malaria parasite densities and species. The slides are used for microscopy trainings, competency assessments and certifications of laboratory personnel in the region. With the assistance of the WHO, the MSB is used for managing a regional Proficiency Testing Scheme for malaria microscopy for national reference laboratories in E8 countries for quality assurance in malaria diagnosis [14]. The facility includes an online interface for registration, result submission, and provision of supportive interaction with participants. The MSB serves as a regional reference facility, strengthening laboratory diagnosis and parasitological testing capability, thereby contributing to the phasing out of any remaining presumptive treatment of malaria based on clinical symptoms alone [14].

Training and certification of laboratory personnel is carried out under the WHO external competency assessment for malaria microscopists (ECAMM) accreditation programme using the MSB. To date more than 100 personnel from laboratories across the E8 region have been trained and certified under this scheme.

#### Malaria genomics in E8

In 2018 the E8 Research Subcommittee identified several research and surveillance areas that should be prioritized to advance malaria elimination in the region. These included routine surveillance of parasite molecular mutations associated with anti-malarial drug resistance, the early detection of histidine rich protein (HRP2/3) gene deletions which can render some types of malaria rapid diagnostic tests (RDT) insensitive, and the provision of overall information on parasite connectivity and sources/sinks across the region. To address these gaps in information, the E8 in collaboration with the University of California San Francisco (UCSF), the NICD in South Africa and Africa Centres for Disease Control and Prevention (CDC) launched the Genomics for Malaria Elimination in E8 countries (GenE8) project in 2021. The project aimed to strengthen malaria parasite surveillance by building capacity in the generation, analysis, and use of parasite genomic data for programmatic decision-making and was instrumental in standardizing assays for genomic work across the region.

Under this project a regional cross-sectional study was conducted collecting blood samples for genomic analysis at selected health facilities of E8 countries in the 2022–2023 transmission season. The data from this survey represented a baseline against which future genomic data can be compared. The project intends to identify use cases of genomic data to support programmatic decision-making, including the monitoring of molecular markers of resistance to anti-malarial drugs and mutations that could affect the functioning of RDTs. To build capacity in the use of parasite genomics in malaria surveillance, a small cohort of suitable laboratory personnel from E8 countries were recruited into a fellowship programme. A series of workshops were conducted to train candidates in malaria parasite genomics including skills in analysing and interpreting malaria parasite genomic data. Participants were also responsible for the standardization of assays and laboratory methods to facilitate a coordinated scale-up of malaria molecular surveillance (MMS) across the region. The research sub-committee and the case management and diagnosis technical working groups collaborated to support these projects.

#### Strengthening vector control and entomology capacity

Vector control through indoor residual spraying (IRS) and distribution of long-lasting insecticidal nets (LLIN) has been the cornerstone for controlling malaria in E8 countries. The effective implementation of IRS requires rigorous planning, skilled human resource and co-ordinated deployment to achieve the WHO optimal coverage and quality of application for maximum impact.

To ensure that IRS is consistently implemented to a high standard, and to avoid the suboptimal coverage and poor quality of IRS that previously contributed to the malaria resurgences during the 2015 malaria season, the vector control technical working group, in collaboration with WHO, developed a standardized training of trainer’s manual and slide deck in 2019. This served as a guide for harmonizing training, implementation, monitoring, and quality assurance for IRS [15]. The use of this manual during in-country trainings has resulted in the adherence to a set of minimum standards linked to indicators that facilitate a like-for-like comparison of IRS coverage across the region. A total of 24 vector control experts (3 per country) were trained as regional trainers of trainers using this guide. This cohort of experts will increase capacity by conducting in-country cascade trainings.

To address the lack of entomological surveillance capacity in the region, the vector control and research sub-committee initiated a regional entomology fellowship in 2017 [16]**.** The fellowship involved a rigorous process to select suitable candidates from each of the E8 countries. The selected fellows underwent intensive training at the University of Witwatersrand (South Africa), Ifakara Health Institute (Tanzania) and Liverpool School of Tropical Medicine (UK). Fellows conducted a 6-month in-country study project to address an identified entomological operational research gap within the national malaria elimination strategies of their country. Each fellow was assigned a mentor from a research institute within their respective countries. An assessment was conducted to evaluate the effectiveness of the fellowship [17]. Based on the recommendations of this assessment a second entomology research fellowship has been initiated to address several entomological research priorities such as vector species identification; vector resting and feeding behaviours; entomological factors associated with residual transmission in elimination settings and monitoring the efficacy of IRS insecticides currently in use in E8 border districts. This work included entomological surveillance to detect the presence of invasive vectors, such as *Anopheles stephensi.* The second fellowship programme comprised of 8 fellows, each nominated by their respective NMCP.

#### Addressing health access and equity barriers to malaria services in E8

The E8 faces disparities in health access among disadvantaged rural and marginalized communities, migrants, and mobile populations [18]. Non-discriminatory services are needed to conform with human rights and gender responsive policies. To identify socio-economic, gender and cultural barriers affecting equity and access to health services among MMPs and in communities living along borders, E8 coordinated a regional assessment, known as the Matchbox Assessment through the community engagement and social behaviour, change and communication technical working group. Groups consisting of male and female MMPs from a homogenous occupational category in each district were selected to participate in focus group discussions using convenience sampling. Focus group discussions were also held with community health workers. Key informant interviews were held with representatives of local health facilities, and community leaders [19]. Based on the assessment the E8 is developing regional community engagement and social behaviour, change and communication elimination guidelines to expand access to health services among underserved populations.

### Regional malaria surveillance

In line with the Global Technical Strategy (GTS) for malaria 2016 – 2030, which considers surveillance as a core intervention [20], the E8 through the SME technical working group adopted a regional surveillance monitoring approach and developed an Epidemic Preparedness and Response (EPR) web application system in 2017. The E8S convenes representatives of Member States and Partners bi-weekly through its platform known as the Situation Room where countries report recent trends on malaria cases, intervention implementation and commodity stock levels. The Situation Room serves as a forum for regular peer-to-peer communication and monitoring of malaria elimination progress in the region. The Situation Room also acts as the E8 cross-border data interface, where monthly counts of malaria cases in border districts are compiled, from which quarterly bulletins on malaria trends are produced. Maps of malaria incidence for 86 border districts are published alongside data on quarterly anomalies of rainfall and temperature to facilitate the interpretation of short-term trends in malaria incidence. The Situation Room also serves as the E8’s regional epidemic preparedness response platform providing countries with recommendations for epidemic response.

To monitor entomological trends and vector control indicators, that are not routinely collected, 16 sentinel sites (2 per country) have been established. These sites collect basic vector control and entomological indicators for standardized reporting at a regional level. Some countries additionally collect vector control quality assurance data.

Table 2 lists malaria indicators that are tracked at regional level. The surveillance, monitoring, and evaluation technical working group has also developed a scorecard as a tool for country accountability towards malaria elimination targets. The scorecard is used to monitor progress using country level indicators on malaria cases and intervention coverages such as indoor residual spraying and net ownership and utilization as well as financial indicators. The inclusion of financing metrics in the E8 Malaria Scorecard enhanced accountability on previously made financial commitments and served as an advocacy tool by highlighting resource gaps to the ministerial sub-committee. The scorecard is presented annually at E8 ministerial committee meetings.

**Table 2:** List of quarterly indicators tracked by country, border district and sentinel site

Epidemic Preparedness and Response (by country)	Border district malaria cases in 86 border districts	Sentinel site surveillance in 16 sites
1. Number of confirmed malaria cases 2. Number of malaria deaths 3. Number of cases classified as local 4. Number of cases classified as imported	1. Number of confirmed malaria cases reported by month 2. % of confirmed malaria cases against suspected cases 3. Number of cases classified as local (frontline countries only) 4. Number of cases classified as imported (frontline countries only) 5. Malaria commodity status 6. Malaria epidemic status (districts that have exceeded outbreak threshold limit values)	1. Total number of cases per health facility 2. Adult vector composition (occurrence and density) 3. Adult vector resting and/or biting behaviour 4. Adult vector insecticide resistance 5. Immature vector aquatic habitats 6. Vector control intervention coverage (IRS, LLINs, Larval source management)

Cases are classified as local or imported based on the patient’s stated recent travel history

## Discussion

Since the inception of the technical working groups, numerous projects have been successfully launched, through the various thematic areas to support malaria elimination efforts in the region. Through these projects efficiencies have been achieved in the programmatic implementation of several key activities. These are. (1) countries now have a Regional Standardized IRS manual for training and implementation; (2) Basic entomological surveillance capability has been set up to support country decision-making on insecticides by utilizing insecticide resistance management strategies for optimal vector control; (3) Health services in border areas are being tailored to become more accessible for resident communities and MMPs; (4) Proficiency and accreditation in malaria microscopy has been optimized and quality assured through the economies of scale that a regional facility could provide; (5) Knowledge and skills in specialist areas such as entomology and parasite genomics have been vastly enhanced through intensive fellowship programmes; (6) EPR and data sharing have been firmly established at a regional level. These initiatives and the associated sharing of best practices are unlikely to have been achieved without the regional approach to technical collaboration that is embodied by the technical working group interactions.

Several challenges exist in translating the technical working group initiatives into progress towards malaria elimination. Whilst elimination remains a priority for the region, there are systemic and structural bottlenecks affecting the pace towards this goal. Firstly, E8 countries face resource constraints preventing the full implementation of national malaria strategic plans. The limited fiscal space in funding malaria programmes arise from low domestic funding commitment towards malaria and low prioritization of malaria due to competing demands to control other diseases as evidenced by the COVID-19 pandemic when resources earmarked for malaria were reprogrammed. Secondly, at the global and regional level, there has been a decline in donor support which is likely to affect implementation of core activities by the NMCPs and risk a reversal of the gains previously made. Of concern is that all four frontline E8 countries will be ineligible for bilateral and multilateral donor support for their malaria programmes from 2024. Among the four countries, three (Botswana, Namibia, and South Africa) are classified as upper-middle income countries and therefore ineligible for direct funding. Eswatini is categorized as low priority by virtue of the low malaria burden and, therefore, funding may be lower than what is needed to pursue elimination goals.

Coupled with this, are the inadequate human resources in some NMCPs through redeployment to other health matters, replacement of staff or the general human resource attrition rates that are common in government institutions in sub-Sahara Africa and that lead to overstretched human resource capacities. This adversely affects the quality of intervention implementation and the uptake of research findings and innovations.

Although agreed to in principle at ministerial level, data sharing has been very slow due to competing data demands from various government bodies and external partnerships, as well as country specific restrictions and regulations around data sharing [18]. However, recently the situation room has seen improvements in participation rates by countries presenting their malaria updates during biweekly meetings, and in making border district data available.

A key question is whether the approach taken by E8 is sustainable and whether it will accelerate elimination. Sustainability of the E8 technical working group approach is threatened by the overall funding environment already described, and by misalignment of the malaria seasonal cycle and the government fiscal cycle, often resulting in lack of funds when they are most needed, for example as the malaria season peaks. Since countries prioritize inland high burden areas at the expense of border districts nearing elimination there is inadequate coordination of cross-border joint planning and pooling of resources which would optimize coverage. Thus, there is a need for advocacy at the highest political level to strengthen the commitment to a regional malaria elimination agenda.

A key lesson learnt is that technical working groups have proven to be an efficient way to initiate new regional projects, share better practices, adapt core interventions, and develop capacity in specialist thematic areas such as genomics and entomology. Technical working groups have also proven to be vital in mobilizing resources for important regional activities as described in this paper. E8 can learn from regional malaria elimination initiatives elsewhere, such as the Asia Pacific Malaria Elimination Network (APMEN). Amongst others, APMEN introduced a vector surveillance and malaria elimination course, a Diploma in applied parasitology and entomology (APMEN, 2020) and an “Online Research Exchange Network Entomology” (ORENE) facility offering specialized entomology capacity building [21]. The ORENE platform also serves as a repository for vector control and entomology resources. These programmes have been sustained with the support of research institutions, governments, and other local collaborators. APMEN and E8 have similar objectives and structures, for example in the running of technical working groups and an exchange of knowledge and experiences could be of benefit to both networks. While APMEN and E8 have their own different governance structures, comparisons can be made between the technical working groups of each. For instance, the APMEN vector control technical working group aims at strengthening entomological surveillance capacity and mobilization of resources for vector control implementation similar to the E8 vector control technical working group. However, APMEN additionally addresses other vector borne diseases in the region. APMEN’s Surveillance and Response technical working group through an integrated health system, aims to increase knowledge in malaria, provision of regional guidance and identifying operational research questions to provide evidence for optimal implementation of tools. This thematic approach aligns with both the E8’s SME-technical working group and the committee on that research that look at malaria surveillance data sharing and setting regional research priorities respectively. On the other hand, although APMEN’s Vivax thematic area focuses mainly on dealing with the threat of this species, it also focusses on malaria case management in line with country and regional guidelines like the diagnosis and case management thematic group in the E8 region.

The E8 can also learn from the Greater Mekong Sub-region which has demonstrated that with high political commitment at ministerial level, funds from International and domestic donors can be raised to support malaria elimination efforts in the region. The regional artemisinin-resistance initiative (RAI) is the largest Global Fund grant and the first catalytic funding established to eliminate malaria in a region [5]. It is primarily funded to mitigate the threat that artemisinin resistance spread may impact on malaria control and elimination globally. As the numerous examples in this paper demonstrate, the Global Fund to fight HIV/AIDS, Tuberculosis and Malaria (GFATM) support for E8 has acted as a catalyst for collective action to eliminate malaria in the region. However, since funding is generally linked to disease burden rather than achieving elimination, E8 is in danger of becoming a victim of its own success. the closer the region gets towards elimination, the harder it will become to attract funding. The stark reality is that without international donor support, the regional elimination approach will likely cease to exist at an operational level as a coordinated and proactive initiative.

## Conclusions

The need for regional collaboration for the effective control and eventual elimination of malaria has long been recognized [22, 23]. To ensure that such collaboration is turned from high level policy declarations into sustained collective action requires permanent structures in which technical experts interact regularly to implement joint initiatives, discuss shared challenges, standardize procedures, adopt best practices and regularly share data on the current malaria situation in each country, including prevailing malaria outbreaks and availability of stocks. The E8 Technical Working Group system embodies such inter-country collaboration of malaria control and elimination activities. The achievements highlighted above would not have been possible without this level of interaction, and without the scale that is provided by a regional collaboration. For example, for each country to develop its own slide bank or its own fellowship programme would not have been feasible. Coordination to limit cross border malaria importation would be unlikely to have achieved sufficiently high priority on an individual country basis. Capacity development for malaria genomic surveillance leverages economies of scale at regional level that would be unlikely on a country-by-country basis. The entomological surveillance fellowship is a model for self-sufficiency in the region which uses existing expertise from local academic and research institutions to provide training and mentorship to increase capacity. Data sharing platforms through the situation room that enables timely responses to challenges being faced by programmes has proved to be an effective tool. These are just some examples of what can only be achieved through regional technical collaboration. Malaria may not have been eliminated yet in any one of the eight countries, but the groundwork for achieving elimination in future, and preparing for future challenges would have been difficult to envisage without this approach. A significant challenge in future will be to maintain the funding that is necessary to sustain collaboration at this intensity, when budgets are stretched, and many priorities compete for limited resources.

## Data Availability

All data and materials presented and generated from the referenced materials. The SADC-MEES materials can be accessed through the link provided https://malariaelimination8.org/sites/default/files/publications/.

## Abbreviations

APMEN: Asia Pacific Malaria Elimination Network
BMGF: Bill and Melinda Gates Foundation
CDC: Centres for Disease Control and Prevention
ECAMM: External Competency Assessment of Malaria Microscopy
E8: Elimination Eight
EPR: Epidemic Preparedness Response
GenE8: Genomics for Malaria Elimination in the E8 countries
GFATM: Global Fund to fight HIV/AIDS, Tuberculosis and Malaria
GTS: Global Technical Strategy for Malaria 2016–2030
HRP2/3: Histidine Rich Protein 2/3
IRS: Indoor residual spraying
LLIN: Long Lasting Insecticidal Net
MMP: Mobile Migrant Population
NICD: National Institute of Communicable Diseases
NMCP: National Malaria Control Programme
ORENE: Online Research Exchange Network Entomology
PPE: Personal Protective Equipment
RDT: Rapid Diagnostic Test
SADC: Southern Africa Development Community
SME: Surveillance, Monitoring and Evaluation
UCSF: University of California San Francisco
VC: Vector Control
WHO: World Health Organization
