# Correction: Malaria elimination and the need for intensive inter-country cooperation: a critical evaluation of regional technical co-operation in Southern Africa

**DOI:** 10.1186/s12936-024-04919-w

**Published:** 2024-04-08

**Authors:** Chadwick H. Sikaala, Bongani Dlamini, Alphart Lungu, Phelele Fakudze, Mukosha Chisenga, Chishala Lukwesa Siame, Nyasha Mwendera, Dumisani Shaba, John M. Chimumbwa, Immo Kleinschmidt

**Affiliations:** 1 SADC Malaria Elimination Eight Secretariat, Windhoek, Namibia; 2 Department of Infectious Disease Epidemiology, London School of Hygiene and Tropical Medicine, London, UK; 3 School of Pathology, Faculty of Health Sciences, University of Witwatersrand, Johannesburg, South Africa

**Correction: Malaria Journal (2024) 23:62** 10.1186/s12936-024-04891-5

Following publication of the article [1], it came to the journal's attention that there was an error in Fig. 3: the image of the figure (the figure apart from the caption) had been erroneously duplicated and showed twice as a result. The figure has since been corrected in the article. The publisher thanks you for reading this erratum and apologizes for any inconvenience caused.
